# Supplementary material for: Knowledge and understanding of cardiovascular disease risk factors in Sierra Leone: a qualitative study of patients’ and community leaders’ perceptions
Source: BMJ Open. 2020 Dec 15;10(12):e038523. doi: 10.1136/bmjopen-2020-038523 (PMC7745312; doi:10.1136/bmjopen-2020-038523)
Supplement: Author's manuscript [file bmjopen-2020-038523.draft_revisions.pdf]

BMJ Open is committed to open peer review. As part of this commitment we make the peer review history of every article we publish publicly available.

When an article is published we post the peer reviewers' comments and the authors' responses online. We also post the versions of the paper that were used during peer review. These are the versions that the peer review comments apply to.

The versions of the paper that follow are the versions that were submitted during the peer review process. They are not the versions of record or the final published versions. They should not be cited or distributed as the published version of this manuscript.

BMJ Open is an open access journal and the full, final, typeset and author-corrected version of record of the manuscript is available on our site with no access controls, subscription charges or pay-per-view fees (http://bmjopen.bmj.com).

If you have any questions on BMJ Open's open peer review process please email info.bmjopen@bmj.com

BMJ Open

Knowledge and understanding of cardiovascular disease risk factors in Sierra Leone: perceptions of patients and community leaders

Journal:	*BMJ Open*
Manuscript ID	bmjopen-2020-038523
Article Type:	Original research
Date Submitted by the Author:	16-Mar-2020

Complete List of Authors:	Ignatowicz, Agnieszka ; University of Birmingham Institute of Applied Health Research<br>Odland, Maria Lisa; University of Birmingham Institute of Applied Health Research, Department of Public Health and Nursing<br>Bockarie, Tahir; University of Warwick Warwick Medical School<br>Wurie, Haja; University of Sierra Leone College of Medicine and Allied Health Sciences<br>Ansumana, Rashid; Mercy Hospital Research Laboratory<br>Kelly, Ann; King's College London, Department of Global Health and Social Medicine<br>Willott, Chris; King's College London, King's Centre for Global Health and Health Partnerships<br>Witham, Miles; AGE Research Group, NIHR Newcastle Biomedical Research Centre, Newcastle University; Newcastle Upon Tyne Hospitals NHS Foundation Trust<br>Davies, Justine ; University of Birmingham Institute of Applied Health Research; King's College London, Centre for Global Health

Keywords:	Health policy < HEALTH SERVICES ADMINISTRATION & MANAGEMENT, PUBLIC HEALTH, QUALITATIVE RESEARCH, Quality in health care < HEALTH SERVICES ADMINISTRATION & MANAGEMENT

SCHOLARONE™
Manuscripts

BMJ

I, the Submitting Author has the right to grant and does grant on behalf of all authors of the Work (as defined in the below author licence), an exclusive licence and/or a non-exclusive licence for contributions from authors who are: i) UK Crown employees; ii) where BMJ has agreed a CC-BY licence shall apply, and/or iii) in accordance with the terms applicable for US Federal Government officers or employees acting as part of their official duties; on a worldwide, perpetual, irrevocable, royalty-free basis to BMJ Publishing Group Ltd ("BMJ") its licensees and where the relevant Journal is co-owned by BMJ to the co-owners of the Journal, to publish the Work in this journal and any other BMJ products and to exploit all rights, as set out in our licence.

The Submitting Author accepts and understands that any supply made under these terms is made by BMJ to the Submitting Author unless you are acting as an employee on behalf of your employer or a postgraduate student of an affiliated institution which is paying any applicable article publishing charge ("APC") for Open Access articles. Where the Submitting Author wishes to make the Work available on an Open Access basis (and intends to pay the relevant APC), the terms of reuse of such Open Access shall be governed by a Creative Commons licence – details of these licences and which Creative Commons licence will apply to this Work are set out in our licence referred to above.

Other than as permitted in any relevant BMJ Author's Self Archiving Policies, I confirm this Work has not been accepted for publication elsewhere, is not being considered for publication elsewhere and does not duplicate material already published. I confirm all authors consent to publication of this Work and authorise the granting of this licence.

For peer review only

Knowledge and understanding of cardiovascular disease risk factors in Sierra Leone: perceptions of patients and community leaders

Agnieszka Ignatowicz[1]*, Maria Lisa Odland[1]*, Tahir Bockarie[2], Haja Wurie[3], Rashid Ansumana[4], Ann H. Kelly[5], Chris Willott[6], Miles D Witham**[7,8] and Justine I Davies**[1,6,9]

*Joint first authorship

** Joint last authorship

[1]Institute of Applied Health Research, College of Medical and Dental Sciences, University of Birmingham, Birmingham, B15 2TT, United Kingdom.

[2]Warwick Medical School, University of Warwick, Coventry, CV4 7AL, United Kingdom.

[3] College of Medicine and Allied Health Sciences, University of Sierra Leone.

[4] School of Community Health Sciences, Njala University, Bo Campus, Sierra Leone.

[5]Department of Global Health and Social Medicine, King's College London, United Kingdom, WC2B 4BG

[6]King's Centre for Global Health and Health Partnerships, King's College London, London, United Kingdom

[7] AGE Research Group, NIHR Newcastle Biomedical Research Centre, Translational Clinical Research Institute, Newcastle University, Newcastle upon Tyne, UK

[8] Newcastle upon Tyne Hospitals Trust, Newcastle upon Tyne, UK

[9]MRC/Wits Rural Public Health & Health Transitions Research Unit (Agincourt), University of the Witwatersrand, Johannesburg, South Africa

Corresponding author:

Maria Lisa Odland, Institute of Applied Health Research, College of Medical and Dental Sciences, University of Birmingham, Birmingham, B15 2TT, United Kingdom. Email: M.L.Odland@bham.ac.uk.

Word count: 5246 without abstract

For peer review only

What is already know about this topic?

- Like many other low-and middle-income countries (LMICs), Sierra Leone is facing an increasing burden of cardiovascular disease risk factors (CVDRF).
- Very little is known about the patient or the community awareness and understanding of CVDRF, and perceptions of barriers and facilitators to accessing care for these conditions.
- This knowledge is essential for designing and implementing cost-effective, equitable interventions that match the needs of the local population.

What does this study add?

- This is the first study exploring patient and community knowledge and understanding of CVDRF in Sierra Leone.
- There is some general knowledge and understanding of CVDRF in the community, but the level and complexity of this knowledge varies widely. Knowledge gaps were found, particularly in relation to coexistence of CVDRF, their consequences and the link between behavioral factors and CVDRF.
- Empowering communities by increasing their knowledge could be an important step in reducing the prevalence of CVDRF and increasing take-up of care for these.

Recommendations for research and policy

- Strategies are needed to improve knowledge and recognition of CVDRF in the community.
- Community led interventions, which could be a cost-effective way to reduce CVDRF burden, need to be tested.

For peer review only

ABSTRACT

Introduction Prevalence of cardiovascular disease risk factors (CVDRF) is increasing, especially in low-income countries. In Sierra Leone, there are no previous studies on the knowledge and the awareness of these conditions in the community. This study aimed to explore the knowledge and understanding of CVDRF, as well as the perceptions of the barriers and facilitators to accessing care for these conditions, amongst patients and community leaders in Sierra Leone.

Methods Participants were recruited from urban and rural Bo District, Sierra Leone for this qualitative study employing semi-structured interviews and focus group discussions with a purposive sample of patients and community leaders. Data were analysed thematically.

Results While participants possessed general knowledge of their conditions, the level and complexity of this knowledge varied widely. There were clear gaps in knowledge regarding the coexistence of CVDRF and their consequences, as well as the link between behavioural factors and CVDRF. An overarching theme from the data was the need to create an understanding and awareness of CVDRF in the community in order to prevent and improve management of these conditions. Cost was also seen as a major barrier to accessing care for CVDRFs.

Conclusions The knowledge gaps identified in this study highlight the need to design strategies and interventions that improve knowledge and recognition of CVDRF in the community. Interventions should specifically consider how to develop and enhance awareness about CVDRF and their consequences. They should also consider how patients seek help and where they access it.

For peer review only

Keywords: CVD risk factors, knowledge and understanding, interviews, focus group discussions, patients, community leaders

For peer review only

INTRODUCTION

Sierra Leone, situated in West-Africa on the Atlantic Ocean, is one of the least developed countries in the world, with a Human Development Index (HDI) of 0.419 (184 of 189 countries).[1 2] The civil war from 1991-2002 disrupted development of the country, including health system infrastructure and services, and the Ebola virus disease in 2013-2016 further inhibited development and health service delivery.[3] However, the country is now recovering from these development challenges, with economic growth and moves towards urbanisation and away from a rural subsistence economy.[4] Economic development and urbanisation are likely to be followed by an increase in behavioural risk factors for cardiovascular disease such as smoking, high salt and sugar intake, alcohol consumption and low levels of exercise.[1] Hence, like many other low-and middle-income countries (LMICs), Sierra Leone is facing an increasing burden of cardiovascular disease risk factors (CVDRF), like diabetes and hypertension,[5 6] and the country is faced with the challenge of dealing with these risk factors and their consequences whilst lacking resources, infrastructure or guidelines.[7 8] A recent publication has also shown that households in LMICs are more likely to experience catastrophic expenditure and impoverishment if they have NCDs, hence the increasing burden of CVDRFs can potentially have major impact on households in this region.[9] We have found that almost 80% (77.1%) of the population in an area in Sierra Leone that is reflective of the country’s population had at least one CVDRF, (Odland et al. "Prevalence and access to care for cardiovascular risk factors in older people in Sierra Leone: A cross-sectional survey") but despite the clear magnitude of the problem, there is little information about knowledge and understanding of CVDRF amongst patients and in the community in Sierra Leone, and indeed, in sub-Saharan Africa more broadly.[10-12]

For peer review only - http://bmjopen.bmj.com/site/about/guidelines.xhtml

Individual and community awareness and knowledge of CVDRF can lead to better prevention and control of these diseases, as knowledge empowers individuals and their communities to act to prevent or manage these conditions.[13-18] While evidence specifically pertaining to CVDRF among people in Sierra Leone is non-existent, a systematic review from Sub-Saharan Africa has shown that lack of knowledge and awareness of CVDRF is strongly associated with low levels of education and rural residency.[12] In Bo, Sierra Leone, where this study was undertaken the majority of the population conforms to these characteristics.[4] Thus, while the prevalence of CVDRF is high knowledge and awareness of CVDRF are likely to be low.[5 6] To enable Sierra Leone to deal effectively with an increasing burden of CVDRF, a better understanding of the patient perceptions-of and experience-with accessing care for CVDRF can help inform the development of interventions that manage and prevent these conditions. Contextually appropriate solutions, capable of grasping the socioeconomic reality of rural life in Sierra Leone, for effective prevention and management of CVDRF are urgently needed. Research on the relationship between community understanding of CVDRF and access to care is therefore key in designing and implementing evidence-based, cost-effective, and equitable interventions. In this analysis, we seek to explore the perspectives of patients and community leaders on CVDRF. Building on the findings from a quantitative component of the study, (Odland et al. "Prevalence and access to care for cardiovascular risk factors in older people in Sierra Leone: A cross-sectional survey") this paper describes knowledge, understanding and practice around CVDRF and perceptions of barriers and facilitators to accessing care.

METHODS

Study setting

For peer review only - http://bmjopen.bmj.com/site/about/guidelines.xhtml

This study was conducted in Bo District, located in the Southern Province of Sierra Leone. The district has rural and urban areas including the country's second largest city after the capital city, Freetown.[4] The distribution of the population is fairly similar to the larger Sierra-Leonean population considering demographic, socioeconomic, and geographical factors like sex, fertility, urban versus rural population, and employment rate.[4] In Bo, Mende is the most common language spoken, but Krio and English are also used. In the most recent census from 2015, there were 575,478 people living in rural areas (66.1%) and 380,307 (33.9%) living in urban areas, mostly Bo City.[4] 17.4% of the population is over 40 years of age.

Sampling and participants

Participants were patients or local community leaders. Patients were interviewed individually, to maximise opportunity for sharing of experiences and perceptions. Community leaders took part in focus group discussions as hierarchical barriers to discussions were felt to be less prominent in community leaders than patients.

We identified patient-participants through a cross-sectional household survey conducted in September-November 2018, which formed the quantitative component of the project. (Odland et al. "Prevalence and access to care for cardiovascular risk factors in older people in Sierra Leone: A cross-sectional survey") Patient participants were randomly selected from those who had answered positively to having CVDRF of hypertension, diabetes or raised cholesterol, or who indicated they were currently taking medicine to treat or prevent heart disease. Participants were only selected if they had previously indicated were happy to be approached for the qualitative interviews. A pre-specified attempt at purposive sampling was done to maximise contrast between men versus women and rural versus urban population.

For peer review only - http://bmjopen.bmj.com/site/about/guidelines.xhtml

Analysis was done iteratively and interviews were conducted until saturation in themes was reached.

For focus groups, community elders/chiefs were approached through local contacts. Snowball sampling was then used to approach participants from amongst the business, religious and traditional healer communities across Bo District. Community members consisted of religious leaders such as imams and pastors, town-criers, and elders, with six to nine members present during each focus group. Two focus groups, one in an urban and one in a rural area, were conducted.

Data collection

The topic guide was developed based on authors' experiences and knowledge of the literature. The questions were designed to explore patients' perceptions of CVDRF, particularly hypertension, diabetes, and their consequences such as stroke or heart attacks, and lived experiences of seeking care and treatment. The interviewer was free to explore various themes that emerged in each individual's interview as well to bring out themes that had emerged during previous interviews. The topic guide was modified for use in the focus group discussions based on the emerging themes from the interviews with patients. The interviews and focus groups were carried out in either Mende or Krio, depending on the language spoken by participants. The interviewers were native Mende or Krio speakers. Interviews and focus groups were recorded, transcribed, and checked by TB and another local transcriber. The interviews and focus group discussions lasted from 45 minutes to 1½ hours and 1½ to 2 hours, respectively.

Data analysis

For peer review only - http://bmjopen.bmj.com/site/about/guidelines.xhtml

Interviews and focus groups were transcribed, anonymised and translated into English by two of the data collectors - one of whom is the co-author on this paper - for subsequent analysis. The transcripts were uploaded into the NVivo programme for coding and analysis.[19] Data were analysed thematically using constant comparison [20] within a modified framework approach [21]. Codes were generated both inductively, from the data, and deductively, focussing on articulations of understanding of CVDRF and perceived barriers to accessing care. A sample of interview transcripts were read to identify the initial set of codes by two co-authors of this paper. This generated an initial coding framework that was discussed in an analysis meeting between members of the research team and then used to code all remaining interview and focus group transcripts. Codes were gradually built into broader categories and final themes through comparison across transcripts, with further discussion amongst all team members.

Research team

The collaboration between UK based and Sierra Leonean researchers, and between medical doctors and social scientists with experience of working in low-income countries and treating patients with CVDRFs, was important for gaining an in-depth understanding of the data and to ensure credibility. During the whole process, the researchers were cognisant of and tried to minimise the effects of their role and background could impact upon the data collection and the analysis. To enhance transferability of the project, full details on the setting, participants, data collection and qualitative analysis are provided in the methods section.

Patient and public involvement statement

Participants were not directly involved in planning the study.

For peer review only - http://bmjopen.bmj.com/site/about/guidelines.xhtml

RESULTS

A total of 37 in depth interviews and two focus groups were undertaken in this study. The demographic characteristics of the study participants are presented in Table 1 and 2 for the in depth interviews and focus groups respectively. The average age for people participating in in depth interviews was 52.23, there was more women (21) than men (16) and a fairly similar distribution between rural vs urban and what language the interviews were conducted in (mende vs krio). In the urban focus group there was only one female, however in the rural focus group there was a fairly similar distribution between men and women. In reporting the findings, we used direct quotes from participants that have been translated into English and anonymized. We tried to translate in a way that closely represented what the person said, as we didn't want to introduce errors into translation.

In summary, while participants possessed some knowledge of their conditions, the level and complexity of this knowledge varied widely. The majority struggled to define CVDRF. Knowledge gaps were found, particularly in relation to coexistence of CVDRF and their consequences. Out of pocket costs were seen as a major barrier to accessing care. An overarching theme from the data was the need to create an understanding and awareness of CVDRF in the community. We discuss these findings in more detail below under three predominant themes of: (1) knowledge and understanding; (2) seeking healthcare; and (3) addressing barriers to access and treatment for CVDRF.

Knowledge and understanding

Diabetes. Participants' understandings of diabetes integrated beliefs about the relationship between "sugar" and "the body", expressed through the ideas that diabetes was caused by "too much sugar".

For peer review only - http://bmjopen.bmj.com/site/about/guidelines.xhtml

When you have too much of sugar in the body, too much sugar is not good for the body. Patient-participant 9, urban area

The sugar (...), the glucose is too much. I know, because we were using it when playing football. But then they said is not good to be in your system, the sugar, too much sugar (...) the sugar should not be plenty in your system. Patient-participant 1, urban area

When discussing diabetes, however, the majority of patient-participants had very limited understanding of what causes diabetes. It was common for the participants to state that sugar directly causes diabetes.

It's a sugar sickness (...) whether it is the ordinary sugar that causes this sickness or where the sugar is coming from, I feel it is sugar that causes this disease [diabetes], (...) that is my own understanding. Patient-participant 22, rural area

Participants talked about diet and cutting down sugar as means of preventing and managing diabetes.

For us in Sierra Leone, rice is our staple food and we have to eat it and they [neighbours] said even the English barbara (sic) rice has enough sugar content in it and we should not eat. (...) when my sugar content is low, I do eat carbohydrate food, fatty food, but I noticed that it is going up, I stopped eating. Patient-participant 17, urban area

For peer review only - http://bmjopen.bmj.com/site/about/guidelines.xhtml

(…) what to eat and what not to eat, because like sugar, or any food that is sweet, if you continue to eat it, you will develop sugar sickness. If we advise people, and they follow it up, maybe they will escape most of those bad conditions. Patient-participant 21, rural area

In the focus groups, rural community leaders elaborated that diabetes would be explained to the patients by health professionals in the same way as the previous quotes described. While leaders could not readily identify the causes of diabetes, some were able to related diet and heredity with an increased risk of developing diabetes.

You get two ways which you acquire diabetes, one is the induced and the other one hereditary. The induced one is like plenty-plenty sugar which we eat, like the sugar in food which we take in. The hereditary is when you acquire from the parents. Community leader, focus group in urban area

However, only a small number of participants mentioned lifestyle changes and exercise, which can prevent or reverse diabetes.

My eating habits and other things. Exercise, my eating habits, I should control these. Patient-participant 32, urban area

Lifestyle, lack of exercise…We just sit-down, we do not exercise, we do not warm-up in the morning, we do not run, or even after work, all the time we are in a car or we are

For peer review only - http://bmjopen.bmj.com/site/about/guidelines.xhtml

on top of motor-bike, so all of that way when we do not exercise it is not well. Community leader, focus group in urban area

Even fewer participants talked about obesity as a factor that can cause diabetes.

They [healthcare professionals] said something on diabetes that if you are overweight will lead to diabetes, but there are people that do not have [large] body that have diabetes. Patient-participant 17, urban area

Most participants in this study could not identify the consequences of diabetes or link diabetes with an increased risk of developing a heart attack or stroke.

Hypertension. Hypertension was perceived to be synonymous with raised or high blood pressure affecting the body, caused by "stress" and "thinking too much", and described in terms of symptoms such as headache, dizziness or "hurting heart".

It is stress, like when you have a lot stress so, like for me so, when you are worried and you are not sleeping well and eating (...) It can be headache or when my heart hurts, the pressure can go up when my heart hurts (...) It's all those things. HSID 146_U

High blood pressure is when the blood in your system, the way it is flowing, because it is regulating the heartbeat, goes higher than the normal. Patient-participant 10, urban area

These understandings of hypertension were also substantiated by community leaders.

For peer review only - http://bmjopen.bmj.com/site/about/guidelines.xhtml

It can be worry, when you worry too much, you trouble yourself too much, your heart becomes spoilt [sic], and it can cause this thing [hypertension]. Community leader, urban focus group

When you have pressure, you have severe headache and when it hurts for long, it may go above 200 or more than that. Community leader, rural focus group

Because stress was viewed as a cause of hypertension, participants talked about stress reduction – worrying less about financial issues, not having to shout at their children or improved sleep – as the main effective way of managing and treating high blood pressure. A small number of participants reported that controlling diet, in particular salt intake, could prevent and help manage the high blood pressure.

We should have control over our diets, that's how we prevent it [high blood pressure]. Patient-participant 15, urban area

If you stop eating salt, you will see the difference. If you always take drugs, the pressure will not affect you at all. Patient-participant 24, rural area

Consequences of CVDRF. Despite limited understanding of causes of CVDRF, some patient-participants talked about the complications of high blood pressure as leading to stroke.

What we know is that stroke is caused by high pressure, if you have high blood pressure and you can't control it, it will result in stoke. Patient-participant 28, rural area

For peer review only - http://bmjopen.bmj.com/site/about/guidelines.xhtml

They say when the pressure is high, this is what leads to hypertension. They say that is what makes one of the hands, or the side [of the body], the stroke, the stoke affects and causes the other side of the body to not function. Patient-participant 25, rural area

However, the majority understood stroke in terms of how the person having a stroke looks and the symptoms experienced.

I have seen people who have a bent month, foot is dead or hands are dead. I cannot tell you exactly what the cause of stroke is. Patient-participant 22, rural area

Only one patient-participant mentioned hearth disease as consequences of hypertension.

Hypertension leads to heart failure. Yes, when people die, I feel that it is heart failure, it is heart failure. Patient-participant 20, urban area

Community leaders acknowledged the relationship and coexistence of CVDRF. For example, hypertension was linked with stroke or diabetes.

Because diabetes can go with hypertension, it's common when you have hypertension, when the sugar is more in the system, that is what causes high blood pressure and hypertension. Community leader, focus group in urban area

This awareness was also expressed by one of the patient-participants, who described hypertension as a "child" of diabetes.

For peer review only - http://bmjopen.bmj.com/site/about/guidelines.xhtml

...diabetes, I feel that it is hypertension's 'pikin' [child, the two are linked], it is their 'pikin', because diabetes has given birth to plenty of 'pikin', one is hypertension. Patient-participant 32, urban area

Seeking healthcare

While participants talked about traditional doctors and medicine, all described seeking and accessing help through the formal healthcare system. Many were of the opinion that diabetes, hypertension or stroke required hospital level care, but recognised that costs associated with transport, consultation, treatment fees and medication were important barriers to seeking help. Those in urban areas preferred to go to healthcare clinics run by foreign healthcare staff because these clinics, in some cases, charged patients only for treatment and medication.

The reason why most people like the [name of the clinic] is because they will not pay for consultation, they will see doctor for free, and when you go you take your card for free. It is the only place that you will see the doctor for free. We thank God for that, because only when you take that paper for ten thousand Leones (approx. £1 sterling), then you will see the doctor. If you have small money at hand, then you will use it to buy your drugs. Patient-participant 36, urban area

Patient-participants in rural areas preferred the nearby public health centres that typically cared for pregnant women, infants and malaria patients, and according to participants, operated a cost-recovery system for some medication. However, the frequency with which medication was out of stock often meant that participants had to seek hospital level care.

For peer review only - http://bmjopen.bmj.com/site/about/guidelines.xhtml

When I cannot travel to [government hospital] there is a clinic here, I have to go there for them to test my pressure, but they don't have drugs, they only check my pressure whether it is high or low. Then I have to go to [government hospital]. Patient-participant 24, rural area

Urban participants talked about seeking help in pharmacies that did not charge for consultations and dispensed medication in the quantities that patients were able to afford.

When I cannot go to hospital, I just go pharmacy. When I go there, they can test me. If it is up [blood pressure], they tell me it is up. They ask which medicine I can take, or which I was taking, then I show them. Community leader, urban focus group

If the sickness [is] serious, we will go to hospital, but for me I go to clinic pharmacy, it's the pharmacy that I will go to if I do not have enough money. Patient-participant 17, urban area

Financial constraints often led patient-participants to delay seeking care. People therefore preferred to go the pharmacy because you were more likely to get the drugs you though you needed, as compared to using money to travel to a hospital, where they ultimately might not provide you with any drugs or treatment. These participants held an opinion that pharmacies were able to give them the drugs that they could afford and provide more caring treatment.

If you do not have enough money to go to government hospital, you go to where they will manage your life, because at the pharmacy, what you have got, is what you will

For peer review only - http://bmjopen.bmj.com/site/about/guidelines.xhtml

buy, the amount you have is the kind drugs you will buy. Patient-participant 11, urban area

For some patient-participants, the costs of seeking help forced them to take medication infrequently to make it last longer or in some cases to forgo treatment altogether. Community leaders elaborated that for those patients who already could not afford consultation, additional costs of medication and transport added to their distress.

Addressing barriers to access and treatment for CVD risk factors

Through the analysis of the data, although costs were highlighted as an issue, lack of knowledge and awareness were singled out as one of the greatest barriers to seeking care for CVDRF. There was a lack of understanding of CVDRF consequences, and when and where to seek care. Moreover, there was a lack of awareness on prevention of conditions. Both patient-participants and community leaders felt that creating understanding and awareness in the community was needed. In the focus groups, community leaders acknowledged the need for better education and prevention.

We need education because they say prevention is better than cure, so when we are able to prevent, then we will reduce the sickness in the community. Community leader, rural focus group

There was a perception that building knowledge, skills and positive attitudes about CVDRF should start at school and involve children and young people.

For peer review only

I would like this education to be extended to schools, because when children are small, you can begin to teach them about this. I think that they will be able to protect themselves so that they will not develop this thing. We need more education, let us not leave it with the adults, let us extend it to various schools. Community leader, rural focus group

Participants agreed that efforts should be increased to raise awareness about CVDRF. However, they noted that despite the magnitude of the CVD burden, there was no specific CVD outreach and sensitisation activities in the community.

These type of sickness, we have not yet seen such activities going on in this community. In case of malaria, they have started supplying the drugs, they advise people to clean their compound, but these other sickness [diabetes, hypertension and stoke], like the one I have now, I have not heard about it. Patient-participant 26, rural area

Community engagement and awareness programmes were seen as important vehicles for increasing knowledge and understanding. Some ideas were mentioned, such as radio, printed information, door-to-door screening and education, and use of public events and forums. Leaders emphasized that any information should be tailored to their community members' needs and communicated in local languages.

They really need to make booklet with pictures so that people will educate themselves, they can do massive public education, awareness rising meetings. Patient-participant 13, urban area

For peer review only - http://bmjopen.bmj.com/site/about/guidelines.xhtml

We need the learning more but let me say, whatever happens, maybe one or two people will be among us that will not hear Krio (...). Let there be somebody that can talk our own language here, Mende, so people can understand in our language. Community leader, rural focus group

Both patient-participants and community leaders agreed that addressing poverty was a key facilitator to increasing access to CVDRF care. They felt that better and more timely access was dependent on addressing the broader social determinants of health, but also health system barriers, such as shortages of drugs.

If people had money, if drugs were available at the hospital, then people would go there as a first point. Like with me, because I have hypertension. If I come to the hospital and I get the treatment I need, I will leave with a positive feeling and refer other people I come across with similar problems. Patient-participant 25, rural area

What I experienced, had it not been the help of chief, I might have died. But he was in Freetown, they rang him, then he brought the drugs. The drugs needed were not in the hospital. So, if it happens that you have no money and the hospital has no drugs, you are going to die. Patient-participant 26, rural area

DISCUSSION

This paper has explored the knowledge of and attitudes towards CVDRF, and perceptions of barriers and facilitators to accessing care amongst patients and community leaders in Sierra Leone. Participants struggled to define diabetes, hypertension or stroke. Knowledge gaps

For peer review only - http://bmjopen.bmj.com/site/about/guidelines.xhtml

were found, particularly around the link between these conditions and their consequences, especially for diabetes. Participants seemed to recognise the increase in the prevalence of various CVDRF in their communities, yet only a few were able to ascribe behavioural risk factors such diet and weight management or regular exercise to CVDRF. There was some knowledge amongst community leaders regarding the relationship and coexistence of CVDRF. Poverty and lack of medications in health facilities were perceived as factors hindering treatment-seeking behaviour for CVDRF conditions. Economic considerations, in particular, were important when seeking help. Patient-participants often talked about seeking help in public health facilities and pharmacies that did not charge for consultation, only choosing hospitals when they judged their symptoms to be more serious. Lack of awareness was seen as the biggest barrier to accessing care and managing CVDRF for patients and community leaders alike.

These knowledge gaps and perceptions should be important considerations when designing and implementing interventions to enhance knowledge of CVDRF in Sierra Leone. They highlight the need to raise awareness about CVDRF consequences, but also behavioural factors and their potential relationships to the development of CVDRF. The limited understanding of the relationships between behavioural factors, different CVDRF conditions and/or their consequences amongst patients and community leaders in this study brings to the fore the need to develop community health education and promotion programmes specifically targeting awareness of these links. To put this to context, in the quantitative component of this study, we found hypertension was present in 49.6%, hypercholesterolemia in 6.7%, and diabetes in 3.5% of the study population. (Odland et al. "Prevalence and access to care for cardiovascular risk factors in older people in Sierra Leone: A cross-sectional survey") Moreover, 25.6% of the population were current or ex (within the last year) smokers and

For peer review only

77.1% had at least one CVDRF. Addressing the burden of CVD in Sierra Leone will require interventions to achieve better awareness, but also support detection and control of these conditions. A recent systematic review on knowledge of CVD risk in sub-Saharan Africa found that the major sources of information for patients included television, radio, newspapers, healthcare professionals, and family members or relatives.[12] Patients and community leaders in our study agreed that implementing and intensifying awareness via radio and other means could be potential platforms to improve knowledge of CVDRF. Additionally, they suggested that the educational programmes could benefit from the development of a more personalized approach tailored to their community members' needs and communicated in local languages. This targeted approach presents an important consideration for development of any educational initiatives. In order to be more successful, it has been suggested that educational programmes should tap into the local views of those directly affected by the CVD conditions.[22] Better approach to CVDRF prevention, as suggested by the community leaders in this study, requires these programmes to start at school and be incorporated into curricula, but this alone may not be sufficient. However, educating on heart health and risk factors, and creating enabling environments where children can learn about warning signs and symptoms, are necessary for prevention of development of CVDRF.

Furthermore, there is a need for better policy to change the health system and accompanying resources to enhance the awareness and practices around CVDRF. Most patients in our study had to pay for medical care and medication out-of-pocket, which constituted a major barrier to accessing care and adherence to long-term or lifelong treatment. Similar findings have been documented in other in low-resourced settings, linking poverty and the lack of policy level interventions to increase access and affordability of services.[23-25] However, some

For peer review only - http://bmjopen.bmj.com/site/about/guidelines.xhtml

successful examples of such interventions exist. MoPoTsyo is a community management scheme in Cambodia that uses a revolving drug fund for CVDRF,[22] and our recent analysis of this program showed substantial and sustained reductions in blood pressure and glucose for those enrolled.[23] To ensure all healthcare needs of individuals are met in countries with underdeveloped health systems, health system policy interventions could consider adopting more community centric models of care.[15]

What is more, patients and community leaders in our study talked about seeking help in local pharmacies. Since community pharmacists seem to be accessible healthcare providers, they may be in position to provide early detection of CVDRF and provide education and counselling when appropriate. Evidence of pharmacists' interventions and the prevention and management of CVD in primary care, particularly in collaboration with other healthcare professionals, demonstrates a positive effect on various patient outcomes.[27]

Community level interventions may also be a possible solution for preventing or managing CVDRF, as has been done previously in Bangladesh.[13] In this study communities were mobilised by applying a participatory learning and action cycle to raise knowledge and awareness of diabetes. Engaging the community can be done more effectively through pre-existing community structures and healthcare providers, alongside deliberate efforts to improve the performance and effectiveness of the healthcare system.[22] Viable community structures exist within Sierra Leone that have the power to deliver better health to community dwellers. There are hierarchical community structures (of Chiefs, Sub-chiefs, and Village Leaders) with regular community meetings, which could be important sites to enhance health behaviour. Finally, as Sierra Leone is developing an NCD plan and its health system, there needs to be focus on CVDRF prevention, monitoring and control. However, any initiatives or

For peer review only - http://bmjopen.bmj.com/site/about/guidelines.xhtml

strategies should be supported by regular monitoring and evaluation by the Ministry of Health in order to evaluate the impact on future CVDRF burden to inform and strengthen the future interventions.

There are several limitations in this study. First, the interviews were only conducted with people who had sought care for conditions and therefore their knowledge may not be reflective of the general population. To ensure the quality of collected data in this study, the interviews and focus group discussions were undertaken by the co-author (TB) who himself is a Sierra Leonean trained in qualitative research. Explanations, clarifications and examples were offered to patients to enhance their understanding of the CVDRF. However, some patients were reluctant to attend interviews because of the fear of receiving bad news about their health. What is more, as participants were not fluent in English, questions were interpreted (into Krio or Mende) and that could have led to additional probing and influenced participants' responses. In some instances, and despite the researcher's clarification and probing, patients were unable to answer the questions. To reduce the effect of these biases in analysis, the authors of this paper undertook member checks to verify whether the interpretations of results reflect what participants actually intended to say, and organized frequent meetings between all study team members in which developing insights were critically discussed. Finally, given the geographical confines of the study setting, the generalizability of these results to other LMIC contexts may be limited. However, the knowledge gaps identified in this study are consistent with findings from other African countries, despite varied levels of socioeconomic growth and literacy levels. For Sierra Leone, however, the transferability of these findings to other similar groups of patients and community leaders may seem justified given variation within our sample for the study.

For peer review only

CONCLUSIONS

This study provides insights into how CVDRF are understood in Sierra Leone, and how the understanding and perception affects treatment-seeking and management for CVD in the community. Knowledge gaps were found, particularly around understanding of the factors that cause CVDRF and their consequences. A lack of awareness was seen as the biggest barrier to accessing care and managing CVDRF in the community. There is need to design strategies and interventions that improve knowledge and recognition of CVDRF, and utilise the perspectives of those affected by these conditions. However, addressing the burden of CVDRF in Sierra Leone will require interventions not only to achieve better awareness, but also support detection and control of these conditions.

Contributors JD, AK, CW and MDW conceived and designed the overall study. JD, AK, CW, TB, HW and RA coordinated data collection and preparation. AI and MLO conducted the analysis, and wrote, and revised the manuscript. JD supervised the analysis, write up, and development of the manuscript. All authors substantively reviewed manuscripts, inputted into revisions, and approved the final manuscript.

Funding Support for the study was given by the Wellcome Trust.

Competing interest AI reports grants from the National Institute of Applied Health Research (NIHR) during the conduct of this study

Patient consent for publication Not required.

Ethical approval Ethical approval was sought and given from the Sierra Leone Ethical and Scientific Review Committee and the BDM Research Ethics sub-committee at King's College London (HR-17/18-7298). Consent to undertake the study was obtained from each village chief or community leader. Consent was obtained from all individuals participating in the study. In the event were participants were illiterate, the consent form was read out to them in the local language and an inked-thumb signature obtained.

Data availability statement Data are not publicly available as consent was not given by participants for this to take place.

Acknowledgements

MDW acknowledges support from the NIHR Newcastle Biomedical Research Centre. Allieu Abu Sheriff DC; Albert Sidikie Sama FM, who helped with the organisation, transcription, and translation of the interviews.

REFERENCES

1 The World Bank. Sierra Leone 2019
Available from: https://data.worldbank.org/country/sierra-leone.
2 UNDP. Human Development Indices and Indicators: 2018 Statistical Update 2018 Available from:

For peer review only - http://bmjopen.bmj.com/site/about/guidelines.xhtml

http://hdr.undp.org/sites/all/themes/hdr_theme/country-notes/SLE.pdf.
3 World Health Organisation. Ebola situation report 30 March. 1th edn. Geneva 2016 Available from: https://www.who.int/csr/disease/ebola/situation-reports/archive/en/.
4 Statistics Sierra Leone. 2015 Population and Housing Census 2015 Available from: https://www.statistics.sl/images/StatisticsSL/Documents/final-results_ 2015_population_and_housing_census.pdf.
5 Sundufu AJ, Bockarie CN, Jacobsen KH. The prevalence of type 2 diabetes in urban Bo, Sierra Leone, and in the 16 countries of the West Africa region. *Diabetes Metab Res Rev* 2017;33(7) doi: 10.1002/dmrr.2904
6 World Health Organisation. WHO STEPs Sierra Leone. 2009
7 Global Burden of Disease Study C. Global, regional, and national incidence, prevalence, and years lived with disability for 301 acute and chronic diseases and injuries in 188 countries, 1990-2013: a systematic analysis for the Global Burden of Disease Study 2013. *Lancet* 2015;386(9995):743-800. doi: 10.1016/S0140-6736(15)60692-4
8 Owolabi M, Miranda JJ, Yaria J, et al. Controlling cardiovascular diseases in low and middle income countries by placing proof in pragmatism. *BMJ Global Health* 2016;1(3):e000105. doi: 10.1136/bmjgh-2016-000105
9 Murphy A, Palafox B, Walli-Attaei M, et al. The household economic burden of non-communicable diseases in 18 countries. *BMJ Glob Health* 2020;5(2):e002040. doi: 10.1136/bmjgh-2019-002040
10 Joshi MD, Ayah R, Njau EK, et al. Prevalence of hypertension and associated cardiovascular risk factors in an urban slum in Nairobi, Kenya: a population-based survey. *BMC Public Health* 2014;14:1177. doi: 10.1186/1471-2458-14-1177
11 Akintunde AA, Akintunde T, Opadijo OG. Knowledge of heart disease risk factors among workers in a Nigerian University: A call for concern. *Niger Med J* 2015;56(2):91-5. doi: 10.4103/0300 1652.150688
12 Boateng D, Wekesah F, Browne JL, et al. Knowledge and awareness of and perception towards cardiovascular disease risk in sub-Saharan Africa: A systematic review. *PLoS One* 2017;12(12):e0189264. doi: 10.1371/journal.pone.0189264
13. Fottrell E, Ahmed N, Morrison J, et al. Community groups or mobile phone messaging to prevent and control type 2 diabetes and intermediate hyperglycaemia in Bangladesh (DMagic): a cluster randomised controlled trial. *Lancet Diabetes Endocrinol* 2019;7(3):200-12. doi: 10.1016/S2213 8587(19)30001-4
14 Land MA, Wu JH, Selwyn A, et al. Effects of a community-based salt reduction program in a regional Australian population. *BMC Public Health* 2016;16:388. doi: 10.1186/s12889-016-3064-3
15 Schneider H, Lehmann U. From Community Health Workers to Community Health Systems: Time to Widen the Horizon? *Health Syst Reform* 2016;2(2):112-18. doi: 10.1080/23288604.2016.1166307
16 Cappuccio FP, Kerry SM, Micah FB, et al. A community programme to reduce salt intake and blood pressure in Ghana [ISRCTN88789643]. *BMC Public Health* 2006;6:13. doi: 10.1186/1471 2458-6-13
17 Aminde LN, Takah N, Ngwasiri C, et al. Population awareness of cardiovascular disease and its risk factors in Buea, Cameroon. *BMC Public Health* 2017;17(1):545. doi: 10.1186/s12889-017 4477-3
18 Wijeysundera HC, Machado M, Farahati F, et al. Association of temporal trends in risk factors and treatment uptake with coronary heart disease mortality, 1994-2005. *Jama* 2010;303(18):1841-7. doi: 10.1001/jama.2010.580
19 QSR International Pty Ltd. Version 12. Vivo qualitative data analysis software, 2018.
20 Glaser BG, Strauss AL. Discovery of Grounded Theory. New York: Routledge 1999.
21 Richie J, Spencer L. Qualitative data analysis for applied policy research. In: Bryman A, Burgess B, eds. Analysing qualitative data. London: Routledge 1994:173–94
22 Murakami H, Phommasack B, Oula R, et al. Revolving drug funds at front-line health facilities in Vientiane, Lao PDR. *Health policy and planning* 2001;16(1):98-106. doi: 10.1093/heapol/16.1.98
23 Nazaneen Nikpour MW, Justine Davies,. An evaluation of an innovative model for care delivery for Cardiovascular Disease Risk factors in the low resource setting of Cambodia (in press). 2020

For peer review only - http://bmjopen.bmj.com/site/about/guidelines.xhtml

For peer review only

Table 1. Demographic characteristics of participants in the individual interviews (n=37)

Age	Average	52.23
Gender	Male	16
	Female	21
Area of living	Rural	18
	Urban	19
Language	Mende	17
	Krio	18
	Mende and Krio	2

Table 2. Characteristics focus groups community members

Area of living	Language	Participants	Gender	Number	Age
Urban	Krio	6 district chiefs; 1 paramount chief for chiefdom	6 males, 1 female	7	40-70 years
Rural	Krio	1 community chief for area; 11 community elders	5 females, 7 males*	12	45-80+ years

For peer review only - http://bmjopen.bmj.com/site/about/guidelines.xhtml

COREQ (COnsolidated criteria for REporting Qualitative research) Checklist

A checklist of items that should be included in reports of qualitative research. You must report the page number in your manuscript where you consider each of the items listed in this checklist. If you have not included this information, either revise your manuscript accordingly before submitting or note N/A.

Topic	Item No.	Guide Questions/Description	Reported on Page No.
**Domain 1: Research team and reflexivity**			
Personal characteristics			
Interviewer/facilitator	1	Which author/s conducted the interview or focus group?	
Credentials	2	What were the researcher's credentials? E.g. PhD, MD	
Occupation	3	What was their occupation at the time of the study?	
Gender	4	Was the researcher male or female?	
Experience and training	5	What experience or training did the researcher have?	
Relationship with participants			
Relationship established	6	Was a relationship established prior to study commencement?	
Participant knowledge of the interviewer	7	What did the participants know about the researcher? e.g. personal goals, reasons for doing the research	
Interviewer characteristics	8	What characteristics were reported about the inter viewer/facilitator? e.g. Bias, assumptions, reasons and interests in the research topic	
**Domain 2: Study design**			
Theoretical framework			
Methodological orientation and Theory	9	What methodological orientation was stated to underpin the study? e.g. grounded theory, discourse analysis, ethnography, phenomenology, content analysis	
Participant selection			
Sampling	10	How were participants selected? e.g. purposive, convenience, consecutive, snowball	
Method of approach	11	How were participants approached? e.g. face-to-face, telephone, mail, email	
Sample size	12	How many participants were in the study?	
Non-participation	13	How many people refused to participate or dropped out? Reasons?	
Setting			
Setting of data collection	14	Where was the data collected? e.g. home, clinic, workplace	
Presence of non-participants	15	Was anyone else present besides the participants and researchers?	
Description of sample	16	What are the important characteristics of the sample? e.g. demographic data, date	
Data collection			
Interview guide	17	Were questions, prompts, guides provided by the authors? Was it pilot tested?	
Repeat interviews	18	Were repeat inter views carried out? If yes, how many?	
Audio/visual recording	19	Did the research use audio or visual recording to collect the data?	
Field notes	20	Were field notes made during and/or after the inter view or focus group?	
Duration	21	What was the duration of the inter views or focus group?	
Data saturation	22	Was data saturation discussed?	
Transcripts returned	23	Were transcripts returned to participants for comment and/or	

For peer review only

Topic	Item No.	Guide Questions/Description	Reported on Page No.
		correction?	
**Domain 3: analysis and findings**			
Data analysis			
Number of data coders	24	How many data coders coded the data?	
Description of the coding tree	25	Did authors provide a description of the coding tree?	
Derivation of themes	26	Were themes identified in advance or derived from the data?	
Software	27	What software, if applicable, was used to manage the data?	
Participant checking	28	Did participants provide feedback on the findings?	
Reporting			
Quotations presented	29	Were participant quotations presented to illustrate the themes/findings? Was each quotation identified? e.g. participant number	
Data and findings consistent	30	Was there consistency between the data presented and the findings?	
Clarity of major themes	31	Were major themes clearly presented in the findings?	
Clarity of minor themes	32	Is there a description of diverse cases or discussion of minor themes?	

Developed from: Tong A, Sainsbury P, Craig J. Consolidated criteria for reporting qualitative research (COREQ): a 32-item checklist for interviews and focus groups. *International Journal for Quality in Health Care*. 2007. Volume 19, Number 6: pp. 349 – 357

Once you have completed this checklist, please save a copy and upload it as part of your submission. DO NOT include this checklist as part of the main manuscript document. It must be uploaded as a separate file.

For peer review only

BMJ Open

Knowledge and understanding of cardiovascular disease risk factors in Sierra Leone: a qualitative study of patients' and community leaders' perceptions

Journal:	*BMJ Open*
Manuscript ID	bmjopen-2020-038523.R1
Article Type:	Original research
Date Submitted by the Author:	02-Jul-2020
Complete List of Authors:	Ignatowicz, Agnieszka ; University of Birmingham Institute of Applied Health Research<br>Odland, Maria Lisa; University of Birmingham Institute of Applied Health Research<br>Bockarie, Tahir; University of Warwick Warwick Medical School<br>Wurie, Haja; University of Sierra Leone College of Medicine and Allied Health Sciences<br>Ansumana, Rashid; Mercy Hospital Research Laboratory<br>Kelly, Ann; King's College London, Department of Global Health and Social Medicine<br>Willott, Chris; King's College London, King's Centre for Global Health and Health Partnerships<br>Witham, Miles; AGE Research Group, NIHR Newcastle Biomedical Research Centre, Newcastle University; Newcastle Upon Tyne Hospitals NHS Foundation Trust<br>Davies, Justine ; University of Birmingham Institute of Applied Health Research; King's College London, Centre for Global Health
**Primary Subject Heading**:	Global health
Secondary Subject Heading:	Diabetes and endocrinology, Global health, Health policy, Cardiovascular medicine, Health services research
Keywords:	Health policy < HEALTH SERVICES ADMINISTRATION & MANAGEMENT, PUBLIC HEALTH, QUALITATIVE RESEARCH

SCHOLARONE™
Manuscripts

I, the Submitting Author has the right to grant and does grant on behalf of all authors of the Work (as defined in the below author licence), an exclusive licence and/or a non-exclusive licence for contributions from authors who are: i) UK Crown employees; ii) where BMJ has agreed a CC-BY licence shall apply, and/or iii) in accordance with the terms applicable for US Federal Government officers or employees acting as part of their official duties; on a worldwide, perpetual, irrevocable, royalty-free basis to BMJ Publishing Group Ltd ("BMJ") its licensees and where the relevant Journal is co-owned by BMJ to the co-owners of the Journal, to publish the Work in this journal and any other BMJ products and to exploit all rights, as set out in our licence.

The Submitting Author accepts and understands that any supply made under these terms is made by BMJ to the Submitting Author unless you are acting as an employee on behalf of your employer or a postgraduate student of an affiliated institution which is paying any applicable article publishing charge ("APC") for Open Access articles. Where the Submitting Author wishes to make the Work available on an Open Access basis (and intends to pay the relevant APC), the terms of reuse of such Open Access shall be governed by a Creative Commons licence – details of these licences and which Creative Commons licence will apply to this Work are set out in our licence referred to above.

Other than as permitted in any relevant BMJ Author's Self Archiving Policies, I confirm this Work has not been accepted for publication elsewhere, is not being considered for publication elsewhere and does not duplicate material already published. I confirm all authors consent to publication of this Work and authorise the granting of this licence.

For peer review only

Knowledge and understanding of cardiovascular disease risk factors in Sierra Leone: a qualitative study of patients' and community leaders' perceptions

Agnieszka Ignatowicz[1]*, Maria Lisa Odland[1]*, Tahir Bockarie[2], Haja Wurie[3], Rashid Ansumana[4], Ann H. Kelly[5], Chris Willott[6], Miles D Witham**[7,8] and Justine I Davies**[1,6,9]

*Joint first authorship

** Joint last authorship

[1]Institute of Applied Health Research, College of Medical and Dental Sciences, University of Birmingham, Birmingham, B15 2TT, United Kingdom.

[2]Warwick Medical School, University of Warwick, Coventry, CV4 7AL, United Kingdom.

[3] College of Medicine and Allied Health Sciences, University of Sierra Leone.

[4] School of Community Health Sciences, Njala University, Bo Campus, Sierra Leone.

[5]Department of Global Health and Social Medicine, King's College London, United Kingdom, WC2B 4BG

[6]King's Centre for Global Health and Health Partnerships, King's College London, London, United Kingdom

[7] AGE Research Group, NIHR Newcastle Biomedical Research Centre, Translational Clinical Research Institute, Newcastle University, Newcastle upon Tyne, UK

[8] Newcastle upon Tyne Hospitals Trust, Newcastle upon Tyne, UK

[9]MRC/Wits Rural Public Health & Health Transitions Research Unit (Agincourt), University of the Witwatersrand, Johannesburg, South Africa

Corresponding author:

Maria Lisa Odland, Institute of Applied Health Research, College of Medical and Dental Sciences, University of Birmingham, Birmingham, B15 2TT, United Kingdom. Email: M.L.Odland@bham.ac.uk.

Word count: 5381 without abstract

For peer review only

ABSTRACT

Objectives Prevalence of cardiovascular disease risk factors (CVDRF) is increasing, especially in low-income countries. In Sierra Leone, there are no previous studies on the knowledge and the awareness of these conditions in the community. This study aimed to explore the knowledge and understanding of CVDRF, as well as the perceptions of the barriers and facilitators to accessing care for these conditions, amongst patients and community leaders in Sierra Leone.

Design Qualitative study employing semi-structured interviews and focus group discussions.

Setting Urban and rural Bo District, Sierra Leone.

Participants Interviews with a purposive sample of 37 patients and two focus groups with six to nine community leaders.

Results While participants possessed general knowledge of their conditions, the level and complexity of this knowledge varied widely. There were clear gaps in knowledge regarding the coexistence of CVDRF and their consequences, as well as the link between behavioural factors and CVDRF. An overarching theme from the data was the need to create an understanding and awareness of CVDRF in the community in order to prevent and improve management of these conditions. Cost was also seen as a major barrier to accessing care for CVDRFs.

Conclusions The knowledge gaps identified in this study highlight the need to design strategies and interventions that improve knowledge and recognition of CVDRF in the community. Interventions should specifically consider how to develop and enhance awareness about CVDRF and their consequences. They should also consider how patients seek help and where they access it.

For peer review only

Keywords: CVD risk factors, knowledge and understanding, interviews, focus group discussions, patients, community leaders

Strengths and limitations of this study

- This is one of the first studies exploring patient and community knowledge and understanding of CVDRF in Sierra Leone.
- Interviews and focus group discussions were undertaken by a researcher who is a Sierra Leonean and trained in qualitative research.
- The study focused on people who had sought care for their conditions and only a small number of community leaders. Therefore, the results may not be representative of the entire community.
- The study was done in one district of Sierra Leone.

For peer review only

INTRODUCTION

Sierra Leone, situated in West-Africa on the Atlantic Ocean, is one of the least developed countries in the world, with a Human Development Index (HDI) of 0.419 (184 of 189 countries).[1 2] The civil war from 1991-2002 disrupted development of the country, including health system infrastructure and services, and the Ebola virus disease in 2013-2016 further inhibited development and health service delivery.[3] However, the country is now recovering from these development challenges, with economic growth and moves towards urbanisation and away from a rural subsistence economy.[4] Economic development and urbanisation are likely to be followed by an increase in behavioural risk factors for cardiovascular disease such as smoking, high salt and sugar intake, alcohol consumption and low levels of exercise. Like in many other sub-Saharan countries, the epidemic of cardiovascular diseases (CVDs) in Sierra Leone is likely to be a real and growing problem[5 6]; more than three quarters of CVD related deaths occur in lower and middle income countries.[6] We and others have found that Sierra Leone is facing an increasing burden of cardiovascular disease risk factors (CVDRF), like diabetes and hypertension,[7-9] and the country is faced with the challenge of dealing with these risk factors and their consequences whilst lacking resources, infrastructure or guidelines.[10-12] However, despite the clear magnitude of the problem, there is little information about knowledge and understanding of CVDRF amongst patients and in the community in Sierra Leone, and indeed, in sub-Saharan Africa more broadly.[13-15]

Individual and community awareness and knowledge of CVDRF can lead to better prevention and control of these diseases, as knowledge empowers individuals and their communities to act to prevent or manage these conditions.[16-21] To enable Sierra Leone to deal effectively with an increasing burden of CVDRF, a better understanding of the patient perceptions-of and experience-with accessing care for CVDRF can help inform the development of interventions

For peer review only - http://bmjopen.bmj.com/site/about/guidelines.xhtml

that manage and prevent these conditions. Contextually appropriate solutions, capable of grasping the socioeconomic reality of rural life in Sierra Leone, for effective prevention and management of CVDRF are urgently needed. Research on the relationship between community understanding of CVDRF and access to care is therefore key in designing and implementing evidence-based, cost-effective, and equitable interventions. In this analysis, we seek to explore the perspectives of patients and community leaders on CVDRF. Building on the findings from a quantitative component of the study[9] this paper describes knowledge, understanding and practice around CVDRF and perceptions of barriers and facilitators to accessing care.

METHODS

Study setting

This study was conducted in Bo District, located in the Southern Province of Sierra Leone. The district has rural and urban areas including the country's second largest city after the capital city, Freetown.[4] The distribution of the population is fairly similar to the larger Sierra-Leonean population considering demographic, socioeconomic, and geographical factors like sex, fertility, urban versus rural population, and employment rate.[4] In Bo, Mende is the most common language spoken, but Krio and English are also used. In the most recent census from 2015, there were 575,478 people living in rural areas (66.1%) and 380,307 (33.9%) living in urban areas, mostly Bo City.[4] 17.4% of the population is over 40 years of age.

Sampling and participants

Participants were either patients known to have cardiovascular diseases or risk factors or local community leaders. Patients were interviewed individually, to maximise opportunity for sharing of experiences and perceptions. Community leaders took part in focus group

For peer review only - http://bmjopen.bmj.com/site/about/guidelines.xhtml

discussions as hierarchical barriers to discussions were felt to be less prominent in community leaders than patients.

We identified patient-participants through a cross-sectional household survey conducted in September-November 2018, which formed the quantitative component of this project.[9] They were selected from those who had answered positively to having CVDRF of hypertension, diabetes, or raised cholesterol, or who indicated they were currently taking medicine to treat or prevent heart disease. To achieve equal representation by sex or rural or urban habitation, we grouped patient-participants by these variables before randomly selecting potential interviewees. Participants were only selected if they had previously indicated were happy to be approached for the qualitative interviews. Analysis was done iteratively and interviews were conducted until saturation in themes was reached.

For focus groups with community leaders, community elders/chiefs were approached through local contacts. Snowball sampling was then used to approach participants from amongst the business, religious and traditional healer communities across Bo District. Community members consisted of religious leaders such as imams and pastors, town-criers, and elders, with six to nine members present during each focus group. Two focus groups, one in an urban and one in a rural area, were conducted.

Data collection

The topic guide was developed based on authors' experiences and knowledge of the literature. The questions were designed to explore patients' perceptions of CVDRF, particularly hypertension, diabetes, and their consequences such as stroke or heart attacks, and lived experiences of seeking care and treatment. The interviewer was free to explore

For peer review only - http://bmjopen.bmj.com/site/about/guidelines.xhtml

various themes that emerged in each individual's interview as well to bring out themes that had emerged during previous interviews. The topic guide was modified for use in the focus group discussions based on the emerging themes from the interviews with patients. The interviews and focus groups were carried out in either Mende or Krio, depending on the language spoken by participants. The interviewers were native Mende or Krio speakers who were trained in qualitative methodologies. Interviews and focus groups were recorded, transcribed, and checked by TB and another local transcriber. The interviews and focus group discussions lasted from 45 minutes to 1½ hours and 1½ to 2 hours, respectively.

Data analysis

Interviews and focus groups were transcribed, anonymised and translated into English by two of the data collectors - one of whom is the co-author on this paper - for subsequent analysis. The transcripts were uploaded into the NVivo programme for coding and analysis.[22] Data were analysed thematically using constant comparison[23] within a modified framework approach.[24] Codes were generated both inductively, from the data, and deductively, focussing on articulations of understanding of CVDRF and perceived barriers to accessing care. A sample of interview transcripts were read to identify the initial set of codes by two co-authors of this paper. This generated an initial coding framework that was discussed in an analysis meeting between members of the research team and then used to code all remaining interview and focus group transcripts. Codes were gradually built into broader categories and final themes through comparison across transcripts, with further discussion amongst all team members. In reporting the findings, direct quotes from participants that have been translated into English and anonymized were used. The quotes were translated in a way that closely represented what the person said and not to introduce errors into translation.

For peer review only - http://bmjopen.bmj.com/site/about/guidelines.xhtml

Research team

The collaboration between UK based and Sierra Leonean researchers, and between medical doctors and social scientists with experience of working in low-income countries and treating patients with CVDRFs, was important for gaining an in-depth understanding of the data and to ensure credibility. During the whole process, the researchers were cognisant of and tried to minimise the effects of their role and background could impact upon the data collection and the analysis. To enhance transferability of the project, full details on the setting, participants, data collection and qualitative analysis are provided in the methods section.

Patient and public involvement statement

Participants were not directly involved in planning the study.

RESULTS

A total of 37 in depth interviews and two focus groups were undertaken in this study. The demographic characteristics of the study participants are presented in Table 1 and 2 for the in-depth interviews and focus groups respectively. The mean age for people participating in in depth interviews was 55.39 (SD 8.52), there was more women (21) than men (16) and a fairly similar distribution between rural vs urban and the language the interviews were conducted in (mende vs krio). In the urban focus group there was only one female, however in the rural focus group there was a fairly similar distribution between men and women.

For peer review only - http://bmjopen.bmj.com/site/about/guidelines.xhtml

Table 1. Demographic characteristics of patient-participants in the individual interviews (n=37)

Age	Average (SD)	55.39 (8.52)
Gender	Male	16
	Female	21
Area of living	Rural	18
	Urban	19
Language	Mende	17
	Krio	18
	Mende and Krio	2

(n=37)

Table 2. Characteristics focus groups with community leaders

Area of living	Language	Participants	Gender	Number	Age
Urban	Krio	6 district chiefs; 1 paramount chief for chiefdom	6 males, 1 female	7	40-70 years
Rural	Krio	1 community chief for area; 11 community elders	5 females, 7 males*	12	45-80+ years

For peer review only

In summary, while participants possessed some knowledge of their conditions, the level and complexity of this knowledge varied widely. The majority struggled to define CVDRF. Knowledge gaps were found, particularly in relation to coexistence of CVDRF and their consequences. Out of pocket costs were seen as a major barrier to accessing care. An overarching theme from the data was the need to create an understanding and awareness of CVDRF in the community. We discuss these findings in more detail below under three predominant themes of: (1) knowledge and understanding; (2) seeking healthcare; and (3) addressing barriers to access and treatment for CVDRF.

For peer review only - http://bmjopen.bmj.com/site/about/guidelines.xhtml

Knowledge and understanding

Diabetes. Participants' understandings of diabetes integrated beliefs about the relationship between "sugar" and "the body", expressed through the ideas that diabetes was caused by "too much sugar".

> *When you have too much of sugar in the body, too much sugar is not good for the body.* Patient-participant 9, urban area

> *The sugar (…), the glucose is too much. I know, because we were using it when playing football. But then they said is not good to be in your system, the sugar, too much sugar (…) the sugar should not be plenty in your system.* Patient-participant 1, urban area

When discussing diabetes, however, the majority of patient-participants had very limited understanding of what causes diabetes. It was common for the participants to state that sugar directly causes diabetes.

> *It's a sugar sickness (…) whether it is the ordinary sugar that causes this sickness or where the sugar is coming from, I feel it is sugar that causes this disease [diabetes], (…) that is my own understanding.* Patient-participant 22, rural area

Participants talked about diet and cutting down sugar as means of preventing and managing diabetes.

> *For us in Sierra Leone, rice is our staple food and we have to eat it and they [neighbours] said even the English barbara (sic) rice has enough sugar content in it*

For peer review only - http://bmjopen.bmj.com/site/about/guidelines.xhtml

and we should not eat. (…) when my sugar content is low, I do eat carbohydrate food, fatty food, but I noticed that it is going up, I stopped eating. Patient-participant 17, urban area

(…) what to eat and what not to eat, because like sugar, or any food that is sweet, if you continue to eat it, you will develop sugar sickness. If we advise people, and they follow it up, maybe they will escape most of those bad conditions. Patient-participant 21, rural area

In the focus groups, rural community leaders elaborated that diabetes would be explained to the patients by health professionals in the same way as the previous quotes described. While leaders could not readily identify the causes of diabetes, some were able to related diet and heredity with an increased risk of developing diabetes.

You get two ways which you acquire diabetes, one is the induced and the other one hereditary. The induced one is like plenty-plenty sugar which we eat, like the sugar in food which we take in. The hereditary is when you acquire from the parents. Community leader, focus group in urban area

However, only a small number of participants mentioned lifestyle changes and exercise, which can prevent or reverse diabetes.

My eating habits and other things. Exercise, my eating habits, I should control these. Patient-participant 32, urban area

For peer review only - http://bmjopen.bmj.com/site/about/guidelines.xhtml

Lifestyle, lack of exercise…We just sit-down, we do not exercise, we do not warm-up in the morning, we do not run, or even after work, all the time we are in a car or we are on top of motor-bike, so all of that way when we do not exercise it is not well. Community leader, focus group in urban area

Even fewer participants talked about obesity as a factor that can cause diabetes.

They [healthcare professionals] said something on diabetes that if you are overweight will lead to diabetes, but there are people that do not have [large] body that have diabetes. Patient-participant 17, urban area

Most participants in this study could not identify the consequences of diabetes or link diabetes with an increased risk of developing a heart attack or stroke.

Hypertension. Hypertension was perceived to be synonymous with raised or high blood pressure affecting the body, caused by "stress" and "thinking too much", and described in terms of symptoms such as headache, dizziness or "hurting heart".

It is stress, like when you have a lot stress so, like for me so, when you are worried and you are not sleeping well and eating (…) It can be headache or when my heart hurts, the pressure can go up when my heart hurts (…) It's all those things. HSID 146_U

High blood pressure is when the blood in your system, the way it is flowing, because it is regulating the heartbeat, goes higher than the normal. Patient-participant 10, urban area

For peer review only - http://bmjopen.bmj.com/site/about/guidelines.xhtml

These understandings of hypertension were also substantiated by community leaders.

> *It can be worry, when you worry too much, you trouble yourself too much, your heart becomes spoilt [sic], and it can cause this thing [hypertension].* Community leader, urban focus group

> *When you have pressure, you have severe headache and when it hurts for long, it may go above 200 or more than that.* Community leader, rural focus group

Because stress was viewed as a cause of hypertension, participants talked about stress reduction – worrying less about financial issues, not having to shout at their children or improved sleep – as the main effective way of managing and treating high blood pressure. A small number of participants reported that controlling diet, in particular salt intake, could prevent and help manage the high blood pressure.

> *We should have control over our diets, that's how we prevent it [high blood pressure].* Patient-participant 15, urban area
>
> *If you stop eating salt, you will see the difference. If you always take drugs, the pressure will not affect you at all.* Patient-participant 24, rural area

Consequences of CVDRF. Despite limited understanding of causes of CVDRF, some patient-participants talked about the complications of high blood pressure as leading to stroke.

For peer review only - http://bmjopen.bmj.com/site/about/guidelines.xhtml

What we know is that stroke is caused by high pressure, if you have high blood pressure and you can't control it, it will result in stoke. Patient-participant 28, rural area

They say when the pressure is high, this is what leads to hypertension. They say that is what makes one of the hands, or the side [of the body], the stroke, the stoke affects and causes the other side of the body to not function. Patient-participant 25, rural area

However, the majority understood stroke in terms of how the person having a stroke looks and the symptoms experienced.

I have seen people who have a bent month, foot is dead or hands are dead. I cannot tell you exactly what the cause of stroke is. Patient-participant 22, rural area

Only one patient-participant mentioned hearth disease as consequences of hypertension.

Hypertension leads to heart failure. Yes, when people die, I feel that it is heart failure, it is heart failure. Patient-participant 20, urban area

Community leaders acknowledged the relationship and coexistence of CVDRF. For example, hypertension was linked with stroke or diabetes.

Because diabetes can go with hypertension, it's common when you have hypertension, when the sugar is more in the system, that is what causes high blood pressure and hypertension. Community leader, focus group in urban area

For peer review only - http://bmjopen.bmj.com/site/about/guidelines.xhtml

This awareness was also expressed by one of the patient-participants, who described hypertension as a "child" of diabetes.

> *...diabetes, I feel that it is hypertension's 'pikin' [child, the two are linked], it is their 'pikin', because diabetes has given birth to plenty of 'pikin', one is hypertension.* Patient-participant 32, urban area

Seeking healthcare

While participants talked about traditional doctors and medicine, all described seeking and accessing help through the formal healthcare system. Many were of the opinion that diabetes, hypertension or stroke required hospital level care, but recognised that costs associated with transport, consultation, treatment fees and medication were important barriers to seeking help. Those in urban areas preferred to go to healthcare clinics run by foreign healthcare staff because these clinics, in some cases, charged patients only for treatment and medication.

> *The reason why most people like the [name of the clinic] is because they will not pay for consultation, they will see doctor for free, and when you go you take your card for free. It is the only place that you will see the doctor for free. We thank God for that, because only when you take that paper for ten thousand Leones (approx. £1 sterling), then you will see the doctor. If you have small money at hand, then you will use it to buy your drugs.* Patient-participant 36, urban area

Patient-participants in rural areas preferred the nearby public health centres that typically cared for pregnant women, infants and malaria patients, and according to participants,

For peer review only - http://bmjopen.bmj.com/site/about/guidelines.xhtml

operated a cost-recovery system for some medication. However, the frequency with which medication was out of stock often meant that participants had to seek hospital level care.

> *When I cannot travel to [government hospital] there is a clinic here, I have to go there for them to test my pressure, but they don't have drugs, they only check my pressure whether it is high or low. Then I have to go to [government hospital].* Patient-participant 24, rural area

Urban participants talked about seeking help in pharmacies that did not charge for consultations and dispensed medication in the quantities that patients were able to afford.

> *When I cannot go to hospital, I just go pharmacy. When I go there, they can test me. If it is up [blood pressure], they tell me it is up. They ask which medicine I can take, or which I was taking, then I show them.* Community leader, urban focus group

> *If the sickness [is] serious, we will go to hospital, but for me I go to clinic pharmacy, it's the pharmacy that I will go to if I do not have enough money.* Patient-participant 17, urban area

Financial constraints often led patient-participants to delay seeking care. People therefore preferred to go the pharmacy because you were more likely to get the drugs you though you needed, as compared to using money to travel to a hospital, where they ultimately might not provide you with any drugs or treatment. These participants held an opinion that pharmacies were able to give them the drugs that they could afford and provide more caring treatment.

For peer review only - http://bmjopen.bmj.com/site/about/guidelines.xhtml

> *If you do not have enough money to go to government hospital, you go to where they will manage your life, because at the pharmacy, what you have got, is what you will buy, the amount you have is the kind drugs you will buy.* Patient-participant 11, urban area

For some patient-participants, the costs of seeking help forced them to take medication infrequently to make it last longer or in some cases to forgo treatment altogether. Community leaders elaborated that for those patients who already could not afford consultation, additional costs of medication and transport added to their distress.

Addressing barriers to access and treatment for CVD risk factors

Through the analysis of the data, although costs were highlighted as an issue, lack of knowledge and awareness were singled out as one of the greatest barriers to seeking care for CVDRF. There was a lack of understanding of CVDRF consequences, and when and where to seek care. Moreover, there was a lack of awareness on prevention of conditions. Both patient-participants and community leaders felt that creating understanding and awareness in the community was needed. In the focus groups, community leaders acknowledged the need for better education and prevention.

> *We need education because they say prevention is better than cure, so when we are able to prevent, then we will reduce the sickness in the community.* Community leader, rural focus group

There was a perception that building knowledge, skills and positive attitudes about CVDRF should start at school and involve children and young people.

For peer review only - http://bmjopen.bmj.com/site/about/guidelines.xhtml

I would like this education to be extended to schools, because when children are small, you can begin to teach them about this. I think that they will be able to protect themselves so that they will not develop this thing. We need more education, let us not leave it with the adults, let us extend it to various schools. Community leader, rural focus group

Participants agreed that efforts should be increased to raise awareness about CVDRF. However, they noted that despite the magnitude of the CVD burden, there was no specific CVD outreach and sensitisation activities in the community.

These type of sickness, we have not yet seen such activities going on in this community. In case of malaria, they have started supplying the drugs, they advise people to clean their compound, but these other sickness [diabetes, hypertension and stoke], like the one I have now, I have not heard about it. Patient-participant 26, rural area

Community engagement and awareness programmes were seen as important vehicles for increasing knowledge and understanding. Some ideas were mentioned, such as radio, printed information, door-to-door screening and education, and use of public events and forums. Leaders emphasized that any information should be tailored to their community members' needs and communicated in local languages.

For peer review only - http://bmjopen.bmj.com/site/about/guidelines.xhtml

They really need to make booklet with pictures so that people will educate themselves, they can do massive public education, awareness rising meetings. Patient-participant 13, urban area

We need the learning more but let me say, whatever happens, maybe one or two people will be among us that will not hear Krio (…). Let there be somebody that can talk our own language here, Mende, so people can understand in our language. Community leader, rural focus group

Both patient-participants and community leaders agreed that addressing poverty was a key facilitator to increasing access to CVDRF care. They felt that better and more timely access was dependent on addressing the broader social determinants of health, but also health system barriers, such as shortages of drugs.

If people had money, if drugs were available at the hospital, then people would go there as a first point. Like with me, because I have hypertension. If I come to the hospital and I get the treatment I need, I will leave with a positive feeling and refer other people I come across with similar problems. Patient-participant 25, rural area

What I experienced, had it not been the help of chief, I might have died. But he was in Freetown, they rang him, then he brought the drugs. The drugs needed were not in the hospital. So, if it happens that you have no money and the hospital has no drugs, you are going to die. Patient-participant 26, rural area

For peer review only - http://bmjopen.bmj.com/site/about/guidelines.xhtml

For peer review only

DISCUSSION

This paper has explored the knowledge of and attitudes towards CVDRF, and perceptions of barriers and facilitators to accessing care amongst patients and community leaders in Sierra Leone. In line with work in other LMICs, participants struggled to define diabetes, hypertension or stroke.[15 25] Knowledge gaps were found, particularly around the link between these conditions and their consequences, especially for diabetes. Participants seemed to recognise the increase in the prevalence of various CVDRF in their communities, yet only a few were able to ascribe behavioural risk factors such diet and weight management or regular exercise to CVDRF. There was some knowledge amongst community leaders regarding the relationship and coexistence of CVDRF. Poverty and lack of medications in health facilities were perceived as factors hindering treatment-seeking behaviour for CVDRF conditions. Economic considerations, in particular, were important when seeking help. This is in line with wider evidence from sub-Saharan Africa, which suggests that widespread poverty affects treatment-seeking and management of CVDRF in the community.[25 26] Patient-participants often talked about seeking help in public health facilities and pharmacies that did not charge for consultation, only choosing hospitals when they judged their symptoms to be more serious. A recent study into the community dynamics surrounding NCDs in Sierra Leone revealed that seeking help from formal health facilities was often the last resort for many patients.[27] However, our findings also indicated that the lack of awareness was seen as the biggest barrier to accessing care and managing CVDRF for patients and community leaders alike.

These knowledge gaps and perceptions should be important considerations when designing and implementing interventions to enhance knowledge of CVDRF in Sierra Leone. They highlight the need to raise awareness about CVDRF consequences, but also behavioural

For peer review only - http://bmjopen.bmj.com/site/about/guidelines.xhtml

factors and their potential relationships to the development of CVDRF. The limited understanding of the relationships between behavioural factors, different CVDRF conditions and/or their consequences amongst patients and community leaders in this study brings to the fore the need to develop community health education and promotion programmes specifically targeting awareness of these links. To put this to context, in the quantitative component of this study, we found hypertension was present in 49.6%, hypercholesterolemia in 6.7%, and diabetes in 3.5% of the study population. Moreover, 25.6% of the population were current or ex (within the last year) smokers and 77.1% had at least one CVDRF.[9] Addressing the burden of CVD in Sierra Leone will require interventions to achieve better awareness, but also support detection and control of these conditions. A recent systematic review on knowledge of CVD risk in sub-Saharan Africa found that the major sources of information for patients included television, radio, newspapers, healthcare professionals, and family members or relatives.[15] Patients and community leaders in our study agreed that implementing and intensifying awareness via radio and other means could be potential platforms to improve knowledge of CVDRF. Additionally, they suggested that the educational programmes could benefit from the development of a more personalized approach tailored to their community members' needs and communicated in local languages. This targeted approach presents an important consideration for development of any educational initiatives. In order to be more successful, it has been suggested that educational programmes should tap into the local views of those directly affected by the CVD conditions.[15] An approach to CVDRF prevention, as suggested by the community leaders in this study, requires these programmes to start at school and be incorporated into curricula, but education about preventing disease alone may not be sufficient. Basic educational skills are important components for achieving health.[28] Fundamental biological knowledge and skills such as reasoning ability are also necessary.

For peer review only - http://bmjopen.bmj.com/site/about/guidelines.xhtml

Effective interventions to enhance CVDRF knowledge in schools and beyond, require deep understanding about the relationships between education and health.

Furthermore, there is a need for better policy to change the health system and accompanying resources to enhance the awareness and practices around CVDRF. Most patients in our study had to pay for medical care and medication out-of-pocket, which constituted a major barrier to accessing care and adherence to long-term or lifelong treatment. Similar findings have been documented in other in low-resourced settings, linking poverty and the lack of policy level interventions to increase access and affordability of services.[28] However, some successful examples of such interventions exist. MoPoTsyo is a community management scheme in that uses a revolving drug fund for CVDRF,[29] and our recent analysis of this program showed substantial and sustained reductions in blood pressure and glucose for those enrolled.[30] To ensure all healthcare needs of individuals are met in countries with underdeveloped health systems, health system policy interventions could consider adopting more community centric models of care.[18]

What is more, patients and community leaders in our study talked about seeking help in local pharmacies. Since community pharmacists seem to be accessible healthcare providers, they may be in position to provide early detection of CVDRF and provide education and counselling when appropriate. Evidence of pharmacists' interventions and the prevention and management of CVD in primary care, particularly in collaboration with other healthcare professionals, demonstrates a positive effect on various patient outcomes.[27]

Community level interventions may also be a possible solution for preventing or managing CVDRF, as has been done previously in Bangladesh.[16] In this study communities were

For peer review only - http://bmjopen.bmj.com/site/about/guidelines.xhtml

mobilised by applying a participatory learning and action cycle to raise knowledge and awareness of diabetes. Engaging the community can be done more effectively through pre-existing community structures and healthcare providers, alongside deliberate efforts to improve the performance and effectiveness of the healthcare system. Viable community structures exist within Sierra Leone that have the power to deliver better health to community dwellers. There are hierarchical community structures (of Chiefs, Sub-chiefs, and Village Leaders) with regular community meetings, which could be important sites to enhance health behaviour. Finally, as Sierra Leone is developing an NCD plan and its health system, there needs to be focus on CVDRF prevention, monitoring and control. However, any initiatives or strategies should be supported by regular monitoring and evaluation by the Ministry of Health in order to evaluate the impact on future CVDRF burden to inform and strengthen the future interventions.

There are several limitations in this study. Interviews were only conducted with people who had sought care for conditions and therefore their knowledge may not be reflective of the general population. Difference in knowledge to the general population may also be found because people with cardiovascular diseases are likely to be better educated and of higher socioeconomic status than those without these diseases. Explanations and examples were offered to patients to enhance their understanding of the CVDRF. However, some patients were reluctant to attend interviews because of the fear of receiving bad news about their health. Although the interviews were done by an interviewer who was fluent in the local language, some of the concepts that were being discussed may not have had a local language equivalent, this could have led to additional probing and influenced participants' responses. In some instances, and despite the researcher's clarification and probing, participants remained were unable to answer the questions. To reduce the effect of these biases in

For peer review only - http://bmjopen.bmj.com/site/about/guidelines.xhtml

analysis, the authors of this paper undertook member checks to verify whether the interpretations of results reflect what participants actually intended to say, and organized frequent meetings between all study team members in which developing insights were critically discussed. Finally, given the geographical confines of the study setting, the generalizability of these results to other LMIC contexts may be limited. However, the knowledge gaps identified in this study are consistent with findings from other African countries, despite varied levels of socioeconomic growth and literacy levels. For Sierra Leone, however, the transferability of these findings to other similar groups of patients and community leaders may seem justified given variation within our sample for the study.

CONCLUSIONS

This study provides insights into how CVDRF are understood in Sierra Leone, and how the understanding and perception affects treatment-seeking and management for CVD in the community. Knowledge gaps were found, particularly around understanding of the factors that cause CVDRF and their consequences. A lack of awareness was seen as the biggest barrier to accessing care and managing CVDRF in the community. There is need to design strategies and interventions that improve knowledge and recognition of CVDRF, and utilise the perspectives of those affected by these conditions. However, addressing the burden of CVDRF in Sierra Leone will require interventions not only to achieve better awareness, but also support detection and control of these conditions.

Contributors JD, AK, CW and MDW conceived and designed the overall study. JD, AK, CW, TB, HW and RA coordinated data collection and preparation. AI and MLO conducted the analysis, and wrote, and revised the manuscript. JD supervised the analysis, write up, and development of the manuscript. All authors substantively reviewed manuscripts, inputted into revisions, and approved the final manuscript.

Funding This work was supported by the Wellcome Trust, grant number 209921/Z/17/Z.

Competing interest AI reports grants from the National Institute of Applied Health Research (NIHR) during the conduct of this study.

Patient consent for publication Not required.

For peer review only - http://bmjopen.bmj.com/site/about/guidelines.xhtml

Ethical approval Ethical approval was sought and given from the Sierra Leone Ethical and Scientific Review Committee and the BDM Research Ethics sub-committee at King's College London (HR-17/18-7298). Consent to undertake the study was obtained from each village chief or community leader. Consent was obtained from all individuals participating in the study. In the event were participants were illiterate, the consent form was read out to them in the local language and an inked-thumb signature obtained.

Data availability statement Data are not publicly available as consent was not given by participants for this to take place.

Acknowledgements MDW acknowledges support from the NIHR Newcastle Biomedical Research Centre. Allieu Abu Sheriff DC; Albert Sidikie Sama FM, who helped with the organisation, transcription, and translation of the interviews.

REFERENES

1. The World Bank. Sierra Leone 2019 [Available from: https://data.worldbank.org.]
2. UNDP. Human Development Indices and Indicators: 2018 Statistical Update 2018 [Available from: http://hdr.undp.org/sites/all/themes/hdr_theme/country-notes/SLE.pdf.]
3. World Health Organisation. Ebola situation report 30 March. 1th edn. Geneva 2016 [Available from: https://www.who.int/csr/disease/ebola/situation-reports/archive/en/.]
4. Statistics Sierra Leone. 2015 Population and Housing Census 2015 [Available from: https://www.statistics.sl/images/StatisticsSL/Documents/final-results_-2015_population_and_housing_census.pdf.]
5. Amegah AK. Tackling the Growing Burden of Cardiovascular Diseases in Sub-Saharan Africa. *Circulation* 2018;138(22):2449-51. doi: 10.1161/CIRCULATIONAHA.118.037367 [published Online First: 2018/12/21]
6. Roth GA, Forouzanfar MH, Moran AE, et al. Demographic and epidemiologic drivers of global cardiovascular mortality. *N Engl J Med* 2015;372(14):1333-41. doi: 10.1056/NEJMoa1406656 [published Online First: 2015/04/02]
7. Sundufu AJ, Bockarie CN, Jacobsen KH. The prevalence of type 2 diabetes in urban Bo, Sierra Leone, and in the 16 countries of the West Africa region. *Diabetes Metab Res Rev* 2017;33(7) doi: 10.1002/dmrr.2904 [published Online First: 2017/04/27]
8. World Health Organisation. WHO STEPs Sierra Leone 2009 [Available from: https://www.who.int/ncds/surveillance/steps/2009_Sierra_Leone_FactSheet_EN.pdf.]
9. Odland ML, Bockarie T, Wurie H, et al. Prevalence and access to care for cardiovascular risk factors in older people in Sierra Leone: a cross-sectional survey. *BMJ Open* 2020;10(9):e038520. doi: 10.1136/bmjopen-2020-038520 [published Online First: 2020/09/11]
10. Global Burden of Disease Study C. Global, regional, and national incidence, prevalence, and years lived with disability for 301 acute and chronic diseases and injuries in 188 countries, 1990-2013: a systematic analysis for the Global Burden of Disease Study 2013. *Lancet* 2015;386(9995):743-800. doi: 10.1016/S0140-6736(15)60692-4 [published Online First: 2015/06/13]
11. Owolabi M, Miranda JJ, Yaria J, et al. Controlling cardiovascular diseases in low and middle income countries by placing proof in pragmatism. *BMJ Glob Health* 2016;1(3) doi: 10.1136/bmjgh-2016-000105 [published Online First: 2016/11/15]
12. Atun R, Davies JI, Gale EAM, et al. Diabetes in sub-Saharan Africa: from clinical care to health policy. *Lancet Diabetes Endocrinol* 2017;5(8):622-67. doi: 10.1016/S2213-8587(17)30181-X [published Online First: 2017/07/10]
13. Joshi MD, Ayah R, Njau EK, et al. Prevalence of hypertension and associated cardiovascular risk factors in an urban slum in Nairobi, Kenya: a population-based survey. *BMC Public Health* 2014;14:1177. doi: 10.1186/1471-2458-14-1177 [published Online First: 2014/11/20]

For peer review only - http://bmjopen.bmj.com/site/about/guidelines.xhtml

14. Akintunde AA, Akintunde T, Opadijo OG. Knowledge of heart disease risk factors among workers in a Nigerian University: A call for concern. *Niger Med J* 2015;56(2):91-5. doi: 10.4103/0300-1652.150688 [published Online First: 2015/04/04]
15. Boateng D, Wekesah F, Browne JL, et al. Knowledge and awareness of and perception towards cardiovascular disease risk in sub-Saharan Africa: A systematic review. *PLoS One* 2017;12(12):e0189264. doi: 10.1371/journal.pone.0189264 [published Online First: 2017/12/13]
16. Fottrell E, Ahmed N, Morrison J, et al. Community groups or mobile phone messaging to prevent and control type 2 diabetes and intermediate hyperglycaemia in Bangladesh (DMagic): a cluster-randomised controlled trial. *Lancet Diabetes Endocrinol* 2019;7(3):200-12. doi: 10.1016/S2213-8587(19)30001-4 [published Online First: 2019/02/09]
17. Land MA, Wu JH, Selwyn A, et al. Effects of a community-based salt reduction program in a regional Australian population. *BMC Public Health* 2016;16:388. doi: 10.1186/s12889-016-3064-3 [published Online First: 2016/05/14]
18. Schneider H, Lehmann U. From Community Health Workers to Community Health Systems: Time to Widen the Horizon? *Health Syst Reform* 2016;2(2):112-18. doi: 10.1080/23288604.2016.1166307 [published Online First: 2016/04/02]
19. Cappuccio FP, Kerry SM, Micah FB, et al. A community programme to reduce salt intake and blood pressure in Ghana [ISRCTN88789643]. *BMC Public Health* 2006;6:13. doi: 10.1186/1471-2458-6-13 [published Online First: 2006/01/26]
20. Aminde LN, Takah N, Ngwasiri C, et al. Population awareness of cardiovascular disease and its risk factors in Buea, Cameroon. *BMC Public Health* 2017;17(1):545. doi: 10.1186/s12889-017-4477-3 [published Online First: 2017/06/07]
21. Wijeysundera HC, Machado M, Farahati F, et al. Association of temporal trends in risk factors and treatment uptake with coronary heart disease mortality, 1994-2005. *JAMA* 2010;303(18):1841-7. doi: 10.1001/jama.2010.580 [published Online First: 2010/05/13]
22. QSR International Pty Ltd. Version 12. Vivo qualitative data analysis software. 2018
23. Glaser BG SA. Discovery of Grounded Theory. New York: Routledge 1999.
24. Richie J SL. Qualitative data analysis for applied policy research. Analysing qualitative data. London: Routledge 1994:173-94.
25. Wekesah FM, Kyobutungi C, Grobbee DE, et al. Understanding of and perceptions towards cardiovascular diseases and their risk factors: a qualitative study among residents of urban informal settings in Nairobi. *BMJ Open* 2019;9(6):e026852. doi: 10.1136/bmjopen-2018-026852 [published Online First: 2019/06/19]
26. BeLue R, Okoror TA, Iwelunmor J, et al. An overview of cardiovascular risk factor burden in sub-Saharan African countries: a socio-cultural perspective. *Global Health* 2009;5:10. doi: 10.1186/1744-8603-5-10 [published Online First: 2009/09/24]
27. Idriss A, Diaconu K, Zou G, et al. Rural-urban health-seeking behaviours for non-communicable diseases in Sierra Leone. *BMJ Glob Health* 2020;5(2):e002024. doi: 10.1136/bmjgh-2019-002024 [published Online First: 2020/03/18]
28. Bukhman G, Mocumbi AO, Atun R, et al. The Lancet NCDI Poverty Commission: bridging a gap in universal health coverage for the poorest billion. *Lancet* 2020 doi: 10.1016/S0140-6736(20)31907-3 [published Online First: 2020/09/18]
29. Murakami H, Phommasack B, Oula R, et al. Revolving drug funds at front-line health facilities in Vientiane, Lao PDR. *Health Policy Plan* 2001;16(1):98-106. doi: 10.1093/heapol/16.1.98 [published Online First: 2001/03/10]
30. Nazaneen Nikpour MW, Justine Davies,. An evaluation of an innovative model for care delivery for Cardiovascular Disease Risk factors in the low resource setting of Cambodia (in press). 2020

For peer review only - http://bmjopen.bmj.com/site/about/guidelines.xhtml

For peer review only

COREQ (COnsolidated criteria for REporting Qualitative research) Checklist

A checklist of items that should be included in reports of qualitative research. You must report the page number in your manuscript where you consider each of the items listed in this checklist. If you have not included this information, either revise your manuscript accordingly before submitting or note N/A.

Topic	Item No.	Guide Questions/Description	Reported on Page No.
**Domain 1: Research team and reflexivity**			
Personal characteristics			
Interviewer/facilitator	1	Which author/s conducted the interview or focus group?	
Credentials	2	What were the researcher's credentials? E.g. PhD, MD	
Occupation	3	What was their occupation at the time of the study?	
Gender	4	Was the researcher male or female?	
Experience and training	5	What experience or training did the researcher have?	
Relationship with participants			
Relationship established	6	Was a relationship established prior to study commencement?	
Participant knowledge of the interviewer	7	What did the participants know about the researcher? e.g. personal goals, reasons for doing the research	
Interviewer characteristics	8	What characteristics were reported about the inter viewer/facilitator? e.g. Bias, assumptions, reasons and interests in the research topic	
**Domain 2: Study design**			
Theoretical framework			
Methodological orientation and Theory	9	What methodological orientation was stated to underpin the study? e.g. grounded theory, discourse analysis, ethnography, phenomenology, content analysis	
Participant selection			
Sampling	10	How were participants selected? e.g. purposive, convenience, consecutive, snowball	
Method of approach	11	How were participants approached? e.g. face-to-face, telephone, mail, email	
Sample size	12	How many participants were in the study?	
Non-participation	13	How many people refused to participate or dropped out? Reasons?	
Setting			
Setting of data collection	14	Where was the data collected? e.g. home, clinic, workplace	
Presence of non-participants	15	Was anyone else present besides the participants and researchers?	
Description of sample	16	What are the important characteristics of the sample? e.g. demographic data, date	
Data collection			
Interview guide	17	Were questions, prompts, guides provided by the authors? Was it pilot tested?	
Repeat interviews	18	Were repeat inter views carried out? If yes, how many?	
Audio/visual recording	19	Did the research use audio or visual recording to collect the data?	
Field notes	20	Were field notes made during and/or after the inter view or focus group?	
Duration	21	What was the duration of the inter views or focus group?	
Data saturation	22	Was data saturation discussed?	
Transcripts returned	23	Were transcripts returned to participants for comment and/or	

For peer review only

Topic	Item No.	Guide Questions/Description	Reported on Page No.
		correction?	
**Domain 3: analysis and findings**			
Data analysis			
Number of data coders	24	How many data coders coded the data?	
Description of the coding tree	25	Did authors provide a description of the coding tree?	
Derivation of themes	26	Were themes identified in advance or derived from the data?	
Software	27	What software, if applicable, was used to manage the data?	
Participant checking	28	Did participants provide feedback on the findings?	
Reporting			
Quotations presented	29	Were participant quotations presented to illustrate the themes/findings? Was each quotation identified? e.g. participant number	
Data and findings consistent	30	Was there consistency between the data presented and the findings?	
Clarity of major themes	31	Were major themes clearly presented in the findings?	
Clarity of minor themes	32	Is there a description of diverse cases or discussion of minor themes?	

Developed from: Tong A, Sainsbury P, Craig J. Consolidated criteria for reporting qualitative research (COREQ): a 32-item checklist for interviews and focus groups. *International Journal for Quality in Health Care*. 2007. Volume 19, Number 6: pp. 349 – 357

Once you have completed this checklist, please save a copy and upload it as part of your submission. DO NOT include this checklist as part of the main manuscript document. It must be uploaded as a separate file.

For peer review only
